# A translational framework of genoproteomic studies for cardiovascular drug discovery

**DOI:** 10.1038/s44325-024-00015-9

**Published:** 2024-08-06

**Authors:** Zhao Yang, Jie V. Zhao, Yue Qi, Xuan Deng, Zhili Ji, Jing Liu

**Affiliations:** 1 Center for Clinical and Epidemiologic Research, Beijing Anzhen Hospital, Capital Medical University, Beijing Institute of Heart, Lung and Blood Vessel Diseases, Beijing, People’s Republic of China; 2 School of Public Health, Li Ka Shing Faculty of Medicine, The University of Hong Kong, 7 Sassoon Road, Pokfulam, Hong Kong SAR, China; 3 Beijing Anzhen Hospital, Capital Medical University, Beijing, China

**Keywords:** Cardiovascular genetics, Interventional cardiology

## Abstract

Cardiovascular drug development has faced significant challenges in recent decades. The emergence of population-scale genome- and proteome-wide data, alongside sophisticated genetic analytical tools like Mendelian randomization and pragmatic target trials, presents an unprecedented chance to identify and validate drug-targeting proteins for cardiovascular disease. However, how to translate these advances into clinical applications remains to be discovered. This study proposes and validates a translational framework that leverages emerging genoproteomic data and cutting-edge causal analysis techniques to address the intricate benefit-risk concerns associated with cardiovascular drug development. Specifically, the framework elucidates underlying biological mechanisms, identifies and validates potential drug-targeting proteins, and explores the unintended side effects, complementary with pragmatic target trials. Moreover, we illustrate the translational framework *via* a step-by-step example alongside practical implementation recommendations for cardiovascular drug discovery. We envision this translational framework as a starting point in advancing multi-omics studies, thereby accelerating cardiovascular drug development.

## Introduction

Cardiovascular disease (CVD), predominantly by ischemic heart disease and stroke, is the leading cause of death globally, contributing to approximately one-third of all deaths^1^. Public health advances in lifestyle and pharmaceutical intervention (e.g., HMGCR^2^ and PCSK9 inhibitors lowering the low-density lipoprotein cholesterol [LDL-C] level^3^) have been reported to avert up to 80% of premature cardiovascular conditions^4^. Nevertheless, the drug development for CVD has encountered stagnation in recent decades^5^, primarily attributed to the lack of clinical efficacy (40%~50%) and safety (~30%)^6–8^. Such a predicament has resulted in a successful rate of <10% for drug development, even after more than a decade of extensive investigation^8^. Of these, targeting non-causal biomarkers and unintended effects on causal biomarkers are the main reasons for the insufficient efficacy and unmanageable side effects. Consequently, a compelling call to act for innovative global approaches to CVD drug solutions was articulated in 2021^9^.

The availability of genetic associations from large-scale biobanks, such as the UK Biobank^10^, deCODE Genetics^11^, Biobank of Japan^12^, China Kadoorie Biobank^13^, FinnGen in Finland^14^, Lifelines in the Netherlands^15^, the Million Veteran Program^16^, and the All of Us Research Program^17^, alongside advances in post-GWAS (i.e., genome-wide association study) analytical techniques (e.g., Mendelian randomization [MR]^18^ and colocalization^19^), presents multiple opportunities^20–24^, such as (1) unraveling the biological mechanisms underpinning the disease causality and identifying risk factors that are causal and pharmaceutically modifiable by manipulating a drug protein target, thereby addressing the non-causal protein targets; (2) discerning the potential side effects and identifying the drug-sensitivity population to mitigate the off-target effect; (3) determining the timing of drug interventions; and (4) predicting the long-term consequences of pharmacological intervention, aiding the drug development. More importantly, drug targets supported by human genetic evidence exhibit a more than 2-fold excess chance of being approved^25,26^ and boost a short drug-discovery life cycle^27^. For instance, in 2021, two-thirds of the US FDA-approved drugs were supported by human genetic evidence involving either gene-encoding protein targets or closely related proteins^28^.

Nevertheless, translating GWAS findings into clinical applications remains challenging, as over 90% of GWAS hits reside in non-coding regions of the genome, solely influencing disease susceptibility or progression^29^. Consequently, these variants’ functional and clinical consequences on putative drug-targeting proteins remain to be discovered^30,31^. One promising avenue for unraveling gene-protein-disease mechanisms is the integration of human genetics and high-throughput proteomics (i.e., genoproteomic). This approach facilitates the disentanglement of complex relationships and provides insights into shared genetic architecture across diseases within a translational framework for drug discovery^24,32,33^, particularly genetic loci situated within or near protein-encoding regions involving *cis*-acting variants that impact the expression of associated proteins directly. Moreover, circulating proteins in human blood, annotated and potentially predicted by protein-coding genes in the human genome, reflect an individual’s dynamic health status and candidate drug targets manipulated by small molecular entities, peptides, or monoclonal antibodies^33,34^. A telling example of such translational approaches is LDL-C-lowering drugs encoded by the PCSK9 gene^35,36^.

This article emphasizes the translational application of genoproteomic data in drug discovery, emphasizing rigorous efforts to address the prevalent challenges of insufficient efficacy and safety. First, we present an analytical, translational framework for cardiovascular drug discovery and validation, which incorporates various Mendelian randomization (MR) methods coupled with several complementary genetic analyses. Then, we illustrate the translational framework by replicating those currently in developed lipid-lowering drug targets encoded by apolipoprotein B (apoB)-containing lipoproteins-lowering *APOB* variants^37^, LDL-C-lowering *LDLR* variants^37^, and lipoprotein(a)-lowering *LPA* variants^38^, along with some general recommendations. We conclude with an in-depth discussion of challenges and remarks on further directions. Herein, we focus on cardiovascular drug discovery, mainly due to the emerging publicly available biobank-scale genetic estimates with sufficient cardiovascular events. Undoubtedly, we can easily extend this translational framework to other diseases.

## Results

### Translational framework of genome-proteome-phenome for drug discovery

**Fig. 1:**
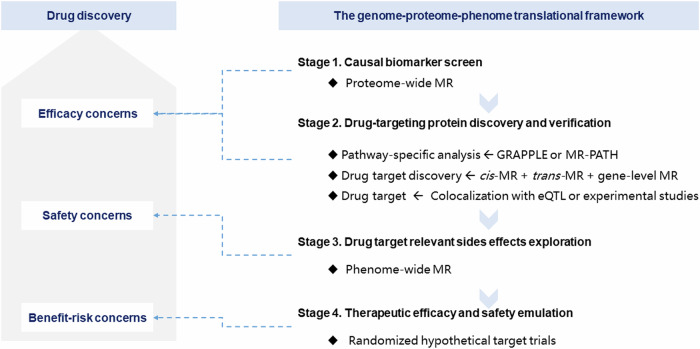
**Scheme of the translational framework for drug discovery.** MR Mendelian randomization.

The presented translational framework integrates the genome, proteome, and phenome in the context of drug discovery by synergistically exploring the interplay between genetic variants, protein levels, and health outcomes using a series of MR analyses and genetic and observational analyses. This framework consists of four stages, as illustrated in Fig. 1, providing a pipeline to bridge the gap between genetic variants and tangible clinical outcomes and facilitating drug discovery.

#### Stage 1. Causal biomarkers screen

**Fig. 2:**
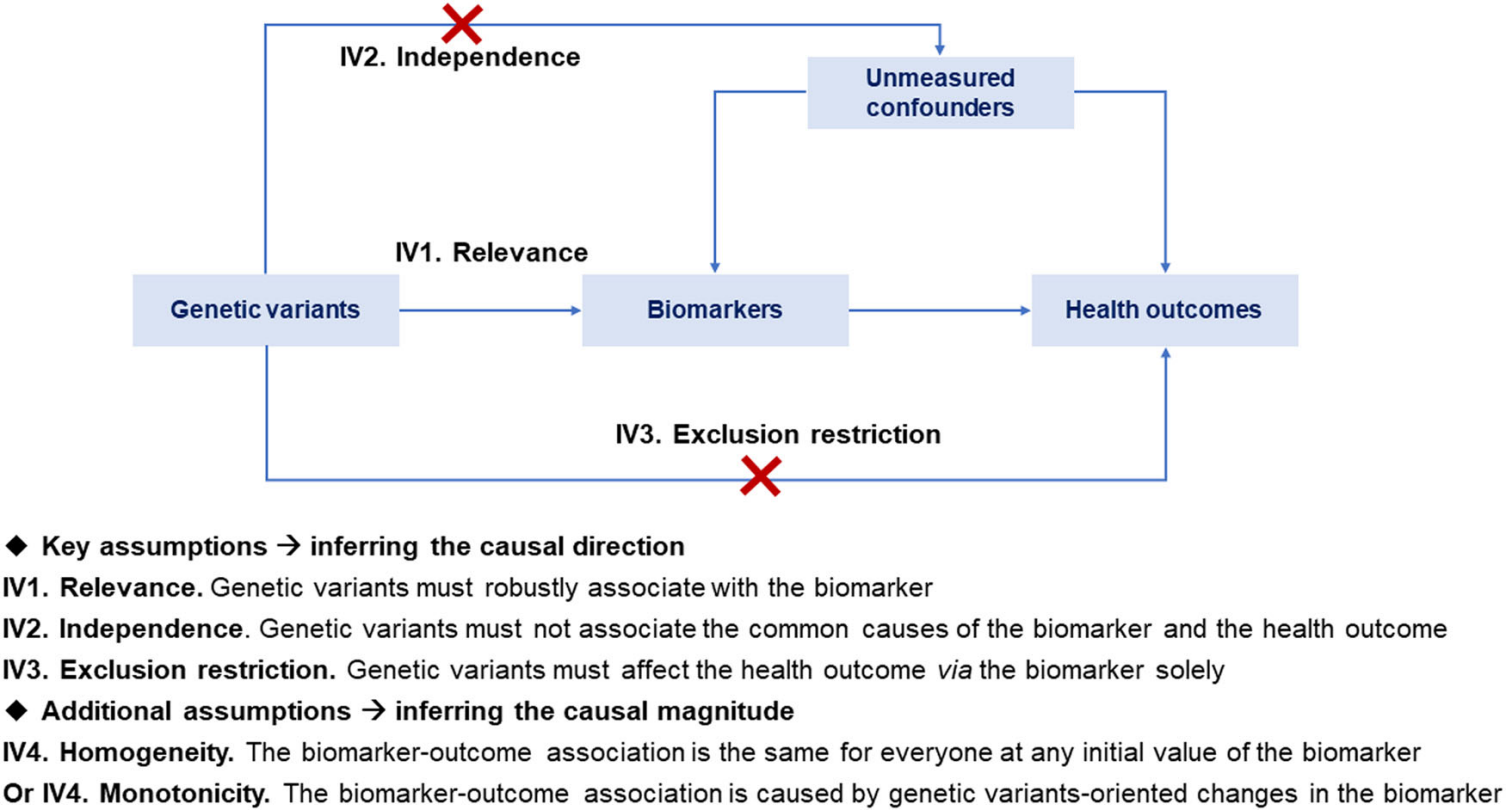
**Principles of Mendelian randomization.** IVs instrumental variables.

This stage involves a proteome-wide MR (i.e., ProMR) analysis, a hypothesis-free design, to pinpoint promising biomarkers causally associated with the health outcome of interest by systematically screening the proteome at a broad scale. We mimicked the downstream effects (i.e., MR estimates, Fig. 2) of candidate biomarkers using genetic variants from large-scale GWAS as instrumental variables (IVs) on health outcomes. Herein, we distinguished biomarkers from drug-targeting proteins, as the former often encapsulates a complex phenotype or a collective trait that objectively reflects the biological processes. In contrast, the latter represents a target capable of modifying the complex phenotype through specific biological mechanisms by appropriate drugs, as extensively discussed in work by Holmes et al.^22^. For instance, biomarkers like LDL-C are well-recognized to be associated with cardiovascular risk, whilst lowering LDL-C typically *via* specific drug-target proteins such as statins^2^ and PCSK9^3^ inhibitors.

**Fig. 3:**
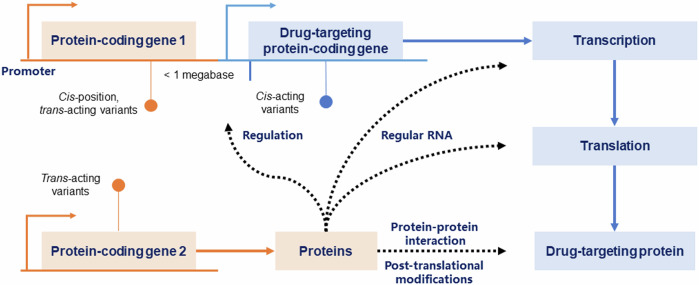
Illustration of *cis*-acting, *trans*-acting, and *cis*-position, *trans*-acting variants affecting the expression and function of a drug-targeting protein. *Cis*-acting variants, located within or near the protein-coding gene, directly influence the expression and function of the drug-targeting protein. In contrast, *trans*-acting variants (including *cis*-position, *trans-*acting variants), resided apart from the protein-coding gene, exert indirect effects, influencing the protein’s expression and function through processes such as RNA-level transcription and translation, post-translational modifications, and protein-protein interactions.

However, considering the mimicked effects derived from genetic estimates exhibited variability and differed from those obtained from randomized controlled trials (RCTs)^39,40^, the IVs used in this stage are not restricted to the independent *cis*-acting variants near or within the protein-coding regions, as illustrated in Fig. 3. In contrast, using IVs at genome-wide significance (*p* *<* 5 × 10^−8^)^41^ or at a less stringent significant level (*p* *<* 1 × 10^−5^)^21,42^ would enhance the power of ProMR as a screening tool. This is particularly useful when only a limited number of IVs are available for the biomarker or when the number of diseases under consideration is small.

Furthermore, given the inherently low prior probability of causality between a biomarker and a health outcome^5–8^, the challenge in this stage lies in how to balance false positive and false negative results from ProMR, which determines the risk of advancing insufficient drug-targeting proteins or overlooks promising drug candidates in late-phase clinical trials^21,42^. To address this problem, we employed a fully automatically estimated *q*-value (i.e., the probability of a null feature being as or more extreme than the observed one) to control the false discovery rate — the expected proportion of how many screened significant biomarkers that are truly null^43–45^. Unlike the false positive rate quantifying the expected proportion of null exposure-outcome associations deemed significant, a *q*-value provides a more clinically meaningful measure. This is particularly relevant for significant biomarkers undergoing subsequent biological verification in the ensuing stage^43^.

Nonetheless, screening the most plausible, or at the very least, statistically robust biomarkers for the health outcome of interest is paramount in cardiovascular drug discovery. This initial step provides crucial evidence indicating that modifying the biomarker can alter the disease’s risk, laying the foundation for subsequent stages of the translational framework.

#### Stage 2. Drug-targeting protein discovery and verification

In Stage 2, we delve into genetic analyses to discern the specific biological mechanisms through which identified causal biomarkers influence disease risk, pinpointing the potential drug-targeting proteins that serve as critical modulators within the relevant pathways.

Though the replication of screened biomarkers in Stage 1 using large samples provides a more straightforward approach to triangulating the robust and consistent biomarker-outcome associations, understanding the underlying downstream biological mechanisms from the biomarker to the health outcome through various genetic analyses is crucial for prioritizing and identifying the putative drug targets. To achieve this, several genetic analyses are employed:

1. Elucidation of possible heterogeneous genetic effects oriented by polygenic IVs across the genome for the biomarker, utilizing methodologies such as Genome-wide mR Analysis under Pervasive PLEitropy (GRAPPLE)^46^ and MR-PATH^47^.

This involves determining upstream regulators or downstream intermediates as putative drug targets. Considering the highly polygenic nature of the complex biomarkers, large-scale GWASs typically identify the associated significant loci across the genome. Further disentangling the underlying biological mechanisms captured by polygenic IVs-oriented heterogeneous effects can help underpin the potential drug targets^46,48^. For example, body mass index (BMI), a polygenic biomarker, has been implicated in the risk of type 2 diabetes (T2D) and coronary heart disease (CHD), as demonstrated in RCTs and MR^49,50^. One plausible pathogenic mechanism from BMI to CHD involves altering the blood glucose levels^50,51^. As such, SGLT1 associated with altered blood glucose levels is expected to influence the risk of obesity and vascular disease^52^. However, genetically mimicking the therapeutic inhibitor of SGLT2 encoding by the SLC5A2 gene on vascular disease remains challenging^53,54^.

(2) Exploration of the putative protein-disease association using *cis*-acting protein quantitative trait loci (*cis*-pQTL) as IVs, supplemented by the inclusion of *trans*-acting variants and *cis*-position, *trans*-acting variants, as appropriate^34^.

The human plasma proteome is comprehensively defined through the analysis and annotation of all potentially protein-coding genes in the human genome^33^, providing a blueprint for the expression and processing of drug-targeting proteins. In GWAS, genetic variants associated with the protein level at genome-wide significance are termed “protein quantitative trait loci (QTLs)”. They are often represented by the most strongly associated single-nucleotide polymorphism. Typically, pQTLs at or near the protein-coding genes in the genome (i.e., *cis*-pQTLs) can directly influence the expression or turnover of the associated protein. In contrast, pQTLs located distinct from the protein-coding gene or on other chromosomes (i.e., *trans*-pQTLs) often exert their influence on the related protein through various indirect processes, such as directly regulating the protein’s transcription-to-translation at the RNA level^33^, as depicted in Fig. 3. Thus, replicating the putative protein-outcome association using *cis*-pQTLs would enhance the credibility of the protein as a drug target^22,23^. For instance, genetic variants within the CEPT gene (Chr 16: bp: 56,961,923-56,985,845; GRCh38^55^) have been previously employed to mimic the therapeutic effect of CEPT inhibitors, illustrating the apoB-mediated effect on CHD rather than an HDL cholesterol-mediated effect^56,57^.

Moreover, incorporating *trans*-pQTLs that influence the related proteins *via* various indirect processes may also help improve the statistical power when only a few *cis*-pQTLs are available and broaden our understanding of the biological implications of the drug-targeting proteins. For instance, recent studies from the UK Biobank Pharma Proteomics Project (UKB-PPP)^24^ show that including *trans*-acting loci into 861 proteins exhibits 1.16 times more detected significant signals than the permuted background.

(3) Verification of putative drug-targeting proteins by examining whether the *cis*-acting variants are shared with the same causal variants as the upstream regulators or downstream intermediates, utilizing colocalization^58,59^ based on gene expression database (e.g., GTEx V8^60^ and eQTLGen^61^) or conducting cell and animal experiments.

Despite substantial progress in studying protein characteristics, translated proteins often undergo complex post-translational processes, leading to their functional exertion through interactions with other proteins^62^. For example, the association of tissue-specific regulatory variants and expression quantitative trait loci (eQTLs) with the health outcome emphasizes the importance of identifying colocalized *cis*-pQTLs that regulate the expression of the associated proteins. Changing the genotypes at this position leads to alternations in the downstream proteins rather than the variants that tend to be physically inherited together (i.e., linkage disequilibrium in which non-causal variants are physically closely correlated with causal variants). Hence, it is crucial to validate putative drug targets by examining whether *cis*-pQTLs are shared with the same causal variants as the upstream regulators or downstream intermediates.

This process is essential for reinforcing the validity of the drug-targeting proteins, though the highly likely colocalization rate (i.e., posterior probability≥0.8) of pQTLs with a disease- or phenotype-related locus was relatively small, ranging from 7.3% to 40%^63–65^, depending on the plasma proteomics and genomics technologies and sample size. Moreover, designing and conducting experimental studies (e.g., CRISPR/Cas9) may enhance the biological implications of intervening in drug-targeting proteins.

#### Stage 3. Drug target relevant side effects exploration

In Stage 3, a phenome-wide MR (PheMR) is employed to investigate the potential side effects of the drug-targeting protein. This critical step aims to comprehensively assess the impact of modifying the protein of interest on a wide range of health outcomes, elucidating the possible unintended consequences and safety profiling, thereby guiding the decision-making process before advancing to late-phase clinical trials.

An illustrative example is found in a recent MR study on ASGR1 inhibitors, where PheMR was employed to investigate the suspected side effects, showing that genetically mimicked ASGR1 inhibitors not only reduced all-cause mortality but increased the risk of liver dysfunction, cholelithiasis, adiposity, and type 2 diabetes^66^. A similar study design was also utilized to evaluate the safety profiling of lipid-modifying medications in Chinese adolescents^67^.

#### Stage 4. Therapeutic efficacy and safety emulation

This stage involves implementing a hypothetically randomized controlled trial (i.e., target trial emulation^68,69^ or pragmatic trials^70,71^) to assess the impact of an intervention, providing a rigorous evaluation of the anticipated benefits and potential risks associated with the drug-targeting protein. Specifically, a hypothetically randomized controlled trial is comparative effectiveness research using big data or large observational databases to emulate a randomized experiment that would answer the specific clinical question of interest when randomized trials are not available^68^.

In contrast to the conventional observational studies, which are susceptible to confounding, selection bias, and reverse causality, the emulation of target trials using available observational data, coupled with appropriate analytical methods, allows for evaluating the benefit-to-risk of the drug-targeting protein^69,72,73^. This can be achieved rigorously by mimicking eligibility criteria, treatment strategies, assignment procedures, outcomes, follow-up, causal contrasts of interest, and statistical methods aligned with the target RCTs. A particularly telling example is the emulation of messenger RNA-based vaccines for preventing COVID-19^74–76^, complemented by large-scale RCTs^77^.

Furthermore, utilizing different study designs susceptible to various bias sources would aid in triangulating the findings from earlier stages, ensuring the observed effects on efficacy and safety of the drug-targeting protein are consistent and replicable^78,79^. Therefore, this stage is pivotal in translating genetic and proteomic findings into actionable evidence. Finally, Table 1 summarizes the key ideas of the methodologies involved in the proposed translational framework.

**Table 1:** Summary of methodologies utilized in the proposed functional, analytical framework for drug target discovery

Methods	Key ideas	Implementation software
Mendelian randomization (MR)^18,96^	Use genetic variants as instrumental variables to estimate the causal effects of modifiable risk factors on the outcome of interest under various assumptions, e.g., independence, relevance, and exclusion restriction assumptions, as well as heterogeneity or monotonicity assumption	https://github.com/vectaport/ivtools https://github.com/MRCIEU/TwoSampleMR https://github.com/cran/MendelianRandomization
Two-sample MR^113^	An application of MR using summary statistics from exposure and outcome genome-wide association studies	https://github.com/MRCIEU/TwoSampleMR; https://github.com/cran/MendelianRandomization
Genome-wide mR Analysis under Pervasive PLEitropy (GRAPPLE)^46^	A three-sample MR method that uses instrumental variables that are strongly and weakly associated with the exposure to analyze the causal effect of a target risk factor with heterogeneous genetic variants and identify possible pleiotropic pathways from data	https://github.com/jingshuw/GRAPPLE
MR-PATH^47^	A two-sample MR that utilizes a latent mixture MR model to exploit effect heterogeneity and clusters instrumental variables dependent on similar genetic estimates	https://github.com/danieliong/MRPATH
*Cis*- and *trans*-acting variants^34^ and drug-target MR	Genetic variants near or within a target gene are defined as *cis*-acting variants, and those far away from a target gene are defined as *trans*-acting variants. Drug-target MR is a variation of MR that uses *cis*-acting variants as instrumental variables.	N.A.
Colocalization^58,59,94^	A Bayesian statistical method that tests whether the same variants drive two or more traits at a particular locus	https://github.com/chr1swallace/coloc https://github.com/cnfoley/hyprcoloc
Phenome-wide MR	An application of MR with various phenotypes or clinical endpoints for a particular exposure of interest	N.A.
Target Trial Emulation^68^	Comparative effectiveness research using the large observational database (big data) to emulate a randomized experiment, i.e., the target experiment or target trial, which would answer the specific clinical question of interest when randomized trials are not available	https://github.com/CausalInference/gfoRmula

### An applied example and some implementing recommendations

To illustrate, we applied the proposed translational framework to replicate the promising lipid-lowering therapeutic targets encoded by *APOB* (triglyceride- and LDL-C-lowering^37^), *LDLR* (LDL-C-lowering^37^), and *LPA* (Lp(a)-lowering^38^) loci to prevent coronary artery disease (CAD) that have been validated in ongoing clinical trials^80–82^. Herein, we focused on genetically mimicked LDL-C-lowering pathways encoded by *APOB*, *LDLR*, and *LPA* loci. Specifically, we used publicly available GWAS summary-level statistics of LDL-C from the Global Lipids Genetics Consortium (GCLC, 1,654,960 individuals)^83^, of CAD from the CARDIoGRAMplusC4D Consortium (60,801 cases and 123,504 controls)^84^, and the 29-year longitudinal data (5735 individuals) from the Chinese Multi-provincial Cohort Study^85,86^. We aligned the effect alleles of variants within *APOB*, *LDLR*, and *LPA* loci as risk-increasing alleles to facilitate the implementation of the proposed framework. More details are presented in the Method section, with the reproducible codes available at https://yangzhao98.github.io/drugTargetScreen/articles/Codes_for_Paper_1.html.

#### Stage 1. Causal biomarkers screen

As positive control examples, we first assessed associations of genetically predicted LDL-C proxied by genome-wide significant SNPs with linkage-disequilibrium (LD) clumped at an *r*^2^ < 0.3 with CAD using inverse-variance weighting two-sample MR estimate with random effects. Like the conventional MR^87^, we exclusively focus on these independent IVs at genome-wide significance without known horizontal pleiotropy *via* the curated and cross-referenced database of PhenoScanner V2^88,89^ to minimize pleiotropic effects and avoid biased MR estimates.

To further ensure the reliability of the selected IVs, we insist on IVs with replication across different studies. We specifically categorized the selected GWAS hits into the following subgroups, as illustrated in Fig. 3.

- *Cis*-pQTLs: Genetic variants reside within or near the protein-coding region in the human genome (e.g., ±10kbp) with the lowest *p*-value;
- *Trans*-pQTLs: Genetic variants distinct from the protein-coding region reside in other parts of the human genome;
- *Cis*-position and *trans*-acting pQTLs: A subset of *trans*-pQTL located within 1 Mb of the protein-coding region in the human genome.

**Fig. 4:**
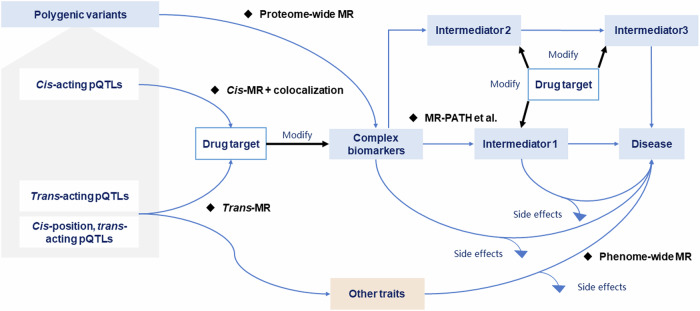
Implementation recommendations of protein quantitative trait loci (pQTLs) as instrumental variables for Mendelian randomization (MR).

Based on the stage-specific purposes of the proposed translational framework, we also provided some practical recommendations for choosing genetic variants as IVs, as summarized in Fig. 4.

**Fig. 5:**
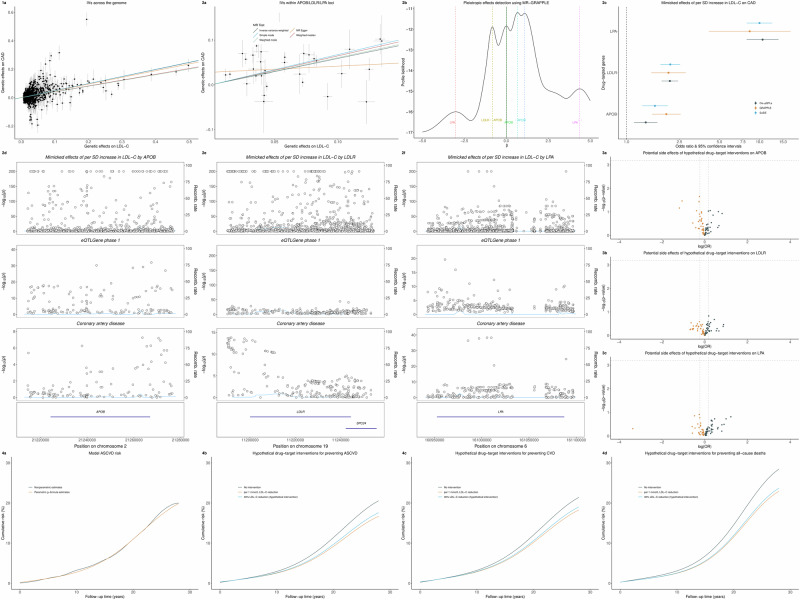
An applied example of the translational framework to replicate the LDL-C-lowering therapeutic targets encoded by *APOB*, *LDLR*, and *LPA* loci on coronary artery disease (CAD). **1a** The scatter plot of genetic effects on LDL-C and CAD using all available genetic variants at genome-wide significance across the genome. **2a** The scatter plot of genetic effects on LDL-C and CAD using all available variants within or near either *APOB*, *LDLR*, or *LPA* loci as instrumental variables (IVs). **2b** The possible pleiotropic effects detected using Genome-wide mR Analysis under Pervasive PLEitropy (GRAPPLE). **2c** Forest plot of the genetically mimicked LDL-C-lowering effects driven by *APOB*, *LDLR*, and *LPA* loci on CAD using *cis*-pQTLs or fine-mapped variants as IVs in Mendelian randomization analyses. **2d**–**f**: Regional plots of colocalization analyses for *APOB*, *LDLR*, *LPA* loci, and expression quantitative trait loci from eQTLGen phase 1 and CAD. **3a**–**c** Volcano plots of the potential side effects of LDL-C-lowering driven by *APOB*, *LDLR*, and *LPA* loci. **4a** The observed and g-formula estimated cumulative atherosclerotic cardiovascular disease (ASCVD) risk based on the Chinese Multi-provincial Cohort Study participants. **4b**–**d**: The estimated cumulative ASCVD, CVD, and all-cause mortality risk with or without hypothetical interventions on lowering LDL-C by modifying *APOB*, *LDLR*, and *LPA*.

Thus, in this stage, the polygenic variants, including *cis*-, *trans*-, and *cis*-position and *trans*-acting pQTLs, were used to screen potential causal biomarkers. We found that genetically mimicked LDL-C per standard deviation (SD) increase was associated with an increased CAD risk (odd ratio [OR] = 1.64; 95% confidence interval [CI]: 1.58–1.70, *p* = 4.51 × 10^−145^), as depicted in Fig. 5-1a, suggesting its capability as a candidate drug target for lowering CAD risk.

#### Stage 2. Drug-targeting protein discovery and verification

Based on the online resource of Ensembl Variant Effect Predictor^90^, supplemented by Genome Aggregation Database^91^ and Functional Annotation of Variants^92^, we limited variants from protein-coding genes of *APOB* (Chr2:21224301-21266945, GRCh37), *LDLR* (Chr19:11200038-11244492), and *LPA* (Chr6:160952515-161087407) to mimic lipid modifying agents. In such a case, genetically mimicked LDL-C per SD increase was associated with an 80% increased CAD risk (OR=1.80, 95% CI: 1.47–2.21, *p* = 1.36 × 10^−8^, Fig. 5-2a**)**.

1. Elucidation of possible heterogeneous genetic effects on LDL-C

We first explored heterogeneous genetic effects (i.e., pleiotropic effects) of these selected variants from protein-coding genes associated with LDL-C levels. Then, we identified possible pleiotropic patterns using GRAPPLE^46^ under a three-sample MR framework, with GWAS summary statistics of LDL-C from GCLC as the selection cohort and those from UK Biobank (343,621 individuals, http://www.nealelab.is/uk-biobank/) as the exposure cohort. We noted multiple possible pleiotropy pathways in the effect of LDL-C with diverging causal directions, as shown in Fig. 5-2b. These suggest different biological mechanisms underlying three candidate druggable targets.

(2) Exploration of *cis*-pQTLs-based LDL-C-CAD associations

We annotated the selected SNPs with Ensembl version 110 (GRCh37). Then, we performed fine-mapping analysis for each gene using the “Sum of Single Effects (SuSiE)” model^93^ to identify a credible set of genetic variants that causally affect LDL-C levels. We termed the druggable SNPs in or near (±10 kbp) region around each gene as *cis*-pQTLs, with others as *trans*-pQTLs. Herein, we didn’t consider the *cis*-position and *trans*-acting variants for simplicity. As expected, genetically mimicked LDL-C per SD increase driven by *APOB* (OR = 1.64; 95% CI: 1.31–2.04, *p* = 1.46 × 10^−5^), *LDLR* (OR = 2.13; 95% CI: 1.80-2.53, *p* = 3.68 × 10^−18^), and *LPA* (OR = 10.0; 95% CI: 8.02-12.45, *p* = 1.71 × 10^−93^) were associated with increased CAD risks (Fig. 5-2c), validating the druggable targets for preventing CAD by lowering LDL-C levels.

(3) Verification of LDL-C-CAD associations with additional use of eQTLs

We further conducted multi-trait colocalization analyses^94^ using a Bayesian divisive clustering algorithm to verify whether the candidate causal SNPs within or near (±10 kbp) each druggable gene associated with downstream expression quantitative trait loci (eQTLs, with summary-level statistics from the eQTLGen Consortium)^61^ and CAD. The genetic effects on LDL-C levels, eQTLs, and CAD were driven by the same causal variants within/near each gene, as depicted in Fig. 5-2d–f.

Moreover, for the *trans*-pQTLs, including *cis*-position and *trans*-acting pQTLs, exploratory analyses are encouraged to unravel potential post-translational modifications, protein-protein interaction networks, or biological pathways involving the drug-targeting protein and other proteins (Fig. 3), alongside the use of additional data with specific molecular phenotypes (e.g., RNA expression and eQTL database^60,61^ with annotated pathway database, e.g., Reactome^95^). For instance, the multi-trait colocalization analyses suggest potential post-translational modifications and protein-protein interactions (Fig. 5-2d–e**)**. Such results emphasize the importance of complementary evidence from *trans*-acting loci for providing additional insights into the biological process involving the drug-target protein.

#### Stage 3. Drug target relevant side effects exploration

Unlike the *cis*-pQTLs used in Stage 2, we implemented hypotheses-free analyses using polygenic variants-based phenome-wide MR for 78 clinical endpoints with ICD-10 diagnoses in UK Biobank (excluding coronary artery disease with ICD-10 code of I25) to explore the potential side effects of the candidate drug targets. There were no significant side effects after controlling the false discovery rate at the 5% level, as depicted in Fig. 5-3a–c. However, such results should be carefully interpreted since we couldn’t exclusively exclude all side effects beyond these 78 identified clinical endpoints.

#### Stage 4. Therapeutic efficacy and safety emulation

Finally, we emulated hypothetical target trials to investigate the preventive effects of mimicking the LDL-C-lowering intervention with the candidate drug targets on atherosclerotic CVD (ASCVD), CVD, and all-cause mortality risk using the 29-year longitudinal study of the Chinese Multi-provincial Cohort Study^85,86^. We found that per 1 mmol/L reduction in LDL-C levels appeared to reduce atherosclerotic CVD risk by 19% (OR = 0.81; 95% CI: 0.65–0.99) among participants with baseline LDL-C ≥ 1.8 mmol/L, consistent with the well-established benefit of approximate one-fifth CVD risk reduction in statins trials^2^. Similar effects were also noted for 40% of LDL-C reduction that mimicked the statins therapies compared with the baseline LDL-C by hypothetically modifying *APOB*, *LDLR*, and *LPA* loci, as depicted in Fig. 5-4a–d.

## Discussion

Despite the notable advances in MR and complementary genetic analyses^19^, which have emerged as powerful tools for cardiovascular drug discovery, challenges persist in applying them to mitigate the insufficient drug safety concerns^6–8^. Previous discussions have extensively covered the potential of these methodologies^20,22,23^, but a consensus on their optimal application to address failures in cardiovascular drug development is yet to be reached. Given the current landscape of genetic-oriented multi-omics studies, with a notable influx of proteomic studies, as documented in http://www.metabolomix.com/a-table-of-all-published-gwas-with-proteomics/, we have centered our efforts on developing this genome-proteome-phenome translational framework. This integrative framework is designed to serve as a pipeline to offer a practical approach to tackle insufficient and unsafety concerns through various post-GWAS analyses of the drug target in late-phase clinical trials. This framework is further reinforced by emulating randomized hypothetical target trials.

However, it is crucial to acknowledge the persisting challenges in this field that require ongoing attention and exploration. The translational framework outlined here heavily relies on MR and, consequently, inherits its limitations in application^96,97^ and interpretation^39,40^. For instance, when evaluating the efficacy and safety of the therapeutic interventions for the drug-targeting protein in clinical practice, MR estimates quantify the life-long effects of the drug-target protein on diseases, which may not accurately capture the short-term therapeutic effect of medication^40^. Additionally, considering that GWAS are often conducted in middle-aged and healthy participants (e.g., UK Biobank^10^, Biobank of Japan^12^, and China Kadoorie Biobank^13^), the genetic associations for the causal biomarkers or the drug-targeting protein are typically biased towards the null, making MR estimates open to selection bias^98,99^. While careful selection of genetic variants as IVs with appropriate analytical methods can mitigate such bias. More importantly, IVs chosen from a middle-aged or older population may differ from those relevant to patients with the disease of interest. Consequently, the identified drug targets may be more pertinent to the onset of the disease rather than its progression among patients. To address this issue, replication of the identified drug target in representative samples of patients, incorporating advanced MR techniques like multivariable MR^100^, becomes imperative. This ensures a more comprehensive and nuanced understanding of the translational implications in the clinical practice.

Another challenge arises from the distribution of proteins within the human body, as presented by the Human Protein Atlas (HPA)^101^. Over two-thirds of proteins reside intracellularly, approximately 28% are membrane-bounded, and ~9% are secreted into the extracellular space. As such, current protein profiling from blood samples may fail to capture crucial intracellular signals^64^, masking potential pleiotropic effects and contributing to failures in drug development, especially concerning efficacy issues. Moreover, current protein-detection techniques, such as Olink and SomaScan, are based on the affinity of the proteins binding to expected targets, only capturing ~30% of proteins encoded in the human genome^65^ based on whole blood samples. Further integrative research combining cutting-edge protein-detection technologies and disease-specific tissues may broaden the spectrum of captured proteins, providing a more comprehensive foundation for drug discovery.

Finally, over 90% of GWAS hits map to the regulation of the associated proteins in non-coding regions of the human genome^102^, primarily due to LD. Typically, LD complicates direct biological and causal inference^103^, yielding unclear downstream effects and challenging the subsequent variant-to-function annotation process^104^. As a result, the selected IVs may fail to effectively mimic the therapeutic effects of the drug target, violating the gene-environment equivalence assumption—the genetic effect from IVs is qualitatively equivalent to the proposed intervention of the drug target^23^. Thus, based on observed patterns of LD and genetic estimates, using statistical fine-mapping (e.g., FINEMAP^105^, CAVIAR^106^, and SuSiE^17,18^) or polygenic priority score^107^ to prioritize the most credible causal variants contributing to disease development within or near the protein-coding gene provides a helpful approach.

Nevertheless, the intricate regulatory downstream network or pathway effects of the non-coding GWAS hits, for instance, post-translational modifications of the drug target occurring at stages of transcription and translation, make the biological implication not easily to be inferred^65^. Thus, identifying regulatory or coregulation pathways of the GWAS hits *via* *cis-* or *trans*-eQTL analysis based on multiple tissue- and cell-level molecular data or functional genomic data may advance their role in regulating network effects contributing to disease development^41^. However, compared to biobank-scale GWAS studies, the sample size of these tissue-specific studies was typically small, limiting their statistical power for detecting significant eQTLs. In such contexts, complementary experimental studies using advanced approaches, such as CRISPR screening in cellular or animal models^108^, provide another practical approach to advancing biological interpretation and drug discovery.

In conclusion, the wealth of large-scale genetic and proteomic data will empower the genome-proteome-phenome translational framework proposed here as a helpful pipeline for cardiovascular drug discovery. Though positive findings from this translational framework do not guarantee the success of drug development, it establishes a practical pipeline and an unprecedented opportunity for drug target screening. We envision this translational framework as a promising starting point for integrating multi-omics to facilitate drug discovery and guide further research toward more effective and safer cardiovascular therapeutics in clinical practice.

## Methods

To illustrate how the proposed translational framework can help prioritize drug targets and accelerate the drug development process, we provided a step-by-step applied example to replicate the promising therapeutic targets of LDL-C encoded by *APOB*^37^, *LDLR*^37^, and *LPA*^38^ loci.

### Data sources

#### GWAS summary statistics of LDL-C

We extracted genetic variants from the aggregated GWAS results from the Global Lipids Genetics Consortium, which included 1,654,960 individuals with an average age of 55 from 201 primary studies, including 99,432 admixed African or African, 146,492 East Asian, 1,320,016 European, 48,057 Hispanic, and 40,963 South Asian participants^83^ as instrumental variables in Mendelian randomization. For each cohort, a standard GWAS protocol (10.21203/rs.3.pex-1687/v1) was used to perform imputation, quality control, and GWAS analysis of low-density lipoprotein cholesterol (LDL-C) with adjustment of age, age^2^, sex, and principal components (PCs) of ancestry. Then, ancestry-specific meta-analyses of LDL-C were performed.

#### GWAS summary statistics of CAD

Meta-analyzed 1000 Genomes-based GWAS results of mainly European (77%), South Asian (India and Pakistan, 13%), and East Asian (China and Korea, 6%) for coronary artery disease (CAD, including 60,801 CAD cases and 123,504 controls) were used^84^. Briefly, minor and major alleles of each contributed GWAS study were identified and imputed by reference to allele frequencies in the pooled populations (all continents) of 1000 Genomes Project phase 1 v3 data. Genetic associations were assessed by the genomic control method, with the overdispersion tissue being adjusted in the inverse variance-weighted fixed-effects meta-analysis. We obtained the aggregated genetic associations for CAD in the applied example.

#### GWAS summary statistics of 78 clinical endpoints in UK Biobank

The UK Biobank recruited approximately 500,000 individuals aged 40–69 years, with 45.6% of men and 94% being self-reported European ancestry from 2006 to 2010 across the United Kingdom^10^. At recruitment, socio-demographic, lifestyle, and health-related factors were collected using questionnaires for all participants after obtaining informed consent. Moreover, biological samples, such as blood, urine, and saliva, were also collected during physical examinations. With these samples, various assays were further examined (e.g., genetic, proteomic, and metabonomic analyses). Follow-up outcomes were obtained from nationwide electronic medical and mortality records. The whole genome-wide analyses of 7221 phenotypes across 6 continental ancestry groups in UK Biobank were conducted by Neale lab (https://www.nealelab.is/uk-biobank) among 361,194 participants (194,174 females and 167,020 males) of British ancestry, with the corresponding genetic estimates adjusting for age, age^2^, inferred sex, age * inferred sex, age^2^ * inferred sex, and the first 20 principal components in sex-combined analyses.

#### Summary statistics of cis- and trans-eQTLs

We obtained summary statistics of *cis*- and *trans*-expression quantitative trait locus (eQTL) from the eQTLGen Consortium, in which 21,684 individuals with 31,684 blood (80.4%) and PBMC (19.6%) samples from 37 datasets were used to detect trait-associated SNPs^61^. Each cohort analyst preprocessed, standardized, and analyzed each contributed data, including genotype expression data preprocessing, PGS calculation and *cis*-eQTL, *trans*-eQTL, and eQTLs mapping. Then, these results from each dataset were meta-analyzed using a weighted Z-score method.

#### Chinese Multi-provincial Cohort Study

The Chinese Multi-provincial Cohort Study (CMCS) is an ongoing longitudinal study in China, which recruited 21,953 participants from multi provinces in China during 1992–1993 (16,811), 1996–1999 (3129), and 2004–2005 (2013)^85,86^. Demographics, lifestyle, and physical characteristics of all participants were collected using a standardized questionnaire modified based on the WHO-MONICA protocol after obtaining informed consent, with clinical measurements being tested in the laboratory using overnight blood samples. All participants were actively followed up for the onset of all fatal and non-fatal acute coronary and stroke events or deaths every 1 to 2 years, supplemented *via* the local disease surveillance systems. Of these participants, up to 5735 CMCS participants aged 35 years or above were actively invited to participate in the in-person follow-up surveys in 2002, 2007, and 2012, and 2020–2023. Moreover, the ASCVD events were defined as acute coronary (including acute myocardial infarction, cardiac arrest, and chronic coronary death) and ischemic stroke events. CVD events were diagnosed based on the criteria of the WHO-MONOCIA project before 2003 and were modified after 2003 following advances in diagnostic technology for myocardial infarction.

### Statistical analyses

Briefly, we selected single nucleotide polymorphism (SNP) at the genome-wide significance of 5 × 10^−8^) with linkage disequilibrium at an *r*^2^ < 0.3 as instrumental variables for LDL-C from the Global Lipids Genetics Consortium https://csg.sph.umich.edu/willer/public/glgc-lipids2021/). We annotated the selected SNPs with Ensembl version 110 (GRCh 37, https://ftp.ensembl.org/pub/grch37/release-110/gff3/homo_sapiens/). We then performed fine-mapping analysis for each gene with the “Sum of Single Effects (SuSiE)” models to identify a credible set of genetic variants that causally affect LDL-C levels. Within the credible set, we termed those within or near (±10 kbp) protein-coding region around each gene as *cis*-acting variants and those far away from the gene as *trans*-acting variants. Herein, we didn’t consider those *cis*-position and *trans*-acting variants for simplicity. Moreover, genetic estimates from the UK Biobank (https://www.nealelab.is/uk-biobank) were employed as the exposure cohort in Genome-wide mR Analysis under Pervasive PLEitropy (GRAPPLE)^46^ in a three-sample MR setting.

Based on the selected SNPs, we obtained MR estimates by meta-analyzing Wald estimates (i.e., genetic association with outcome divided by genetic association with exposure) using inverse variance weighting with fixed effects for three SNPs or fewer and random effects for four SNPs or above^109^. Moreover, weighted median^110^, weighted mode, simple mode MR^111^, and MR Egger regression^112^ were performed to quantify the uncertainty of LDL-C-CAD associations, allowing for violation of IV assumptions. Then, we performed colocation analyses under the hypothesis prioritization for muti-trait colocalization framework to assess the posterior probability of a shared variant within or near (±10 kbp) of the druggable gene, expression quantitative traits (eQTLs) and CAD using the default settings of prior probabilities. We extracted summary statistics of eQTLs from the eQTLGen Consortium (https://www.eqtlgen.org/phase1.html)^61^. A posterior probability greater than 0.8 was considered strong evidence of colocalization^94^. Next, we conducted a phenome-wide MR using 78 clinical endpoints with ICD-10 diagnoses in the UK Biobank (https://www.nealelab.is/uk-biobank). Before the analyses, we transformed the genetic estimates derived from the linear mixed model to odds ratio (OR) for binary outcomes, adhering to the formula provided in the MRC IEU UK Biobank GWAS pipeline, version 18/01/2019 (https://data.bris.ac.uk/data/dataset/). Finally, we explicitly emulated hypothetical target trials using the 29-year follow-up data from CMCS to investigate the genetically mimicked LDL-C-lowering effects encoded by *APOB*, *LDLR*, and *LPA* loci on ASCVD, CVD, and all-cause mortality. Table 2 describes the protocol of the target trial and the target trial emulation.

**Table 2:** Specification and emulation of a target trial of LDL-C-lowering interventions to prevent cardiovascular disease and all-cause mortality

Protocol component	Target trial specification	Target trial emulation
Eligibility criteria	Aged 35 years or older recruited between January 1, 1992 and December 31, 1993 No history of cardiovascular disease at the baseline Triglyceride < 4.52 mmol/L (400 mg/dL) at baseline The baseline is defined as the recruitment date of each participant	Same as for the target trial. We defined a history of cardiovascular disease as a composite outcome comprising heart disease and stroke before the recruitment date in the Chinese Multi-provincial Cohort Study.
Treatment strategies	(1) Initiation of a targeted therapy mimicking effects of per 1 mmol/L LDL-C reduction or 40% reduction comparing the baseline level among participants with baseline LDL-C level greater than 1.8 mmol/L. (2) No intervention	Same as for the target trial.
Treatment assignment	Participants are randomly assigned to an intervention strategy at baseline	We implemented the hypothetical intervention for all eligible participants when the condition is met at baseline and attempted to emulate randomization by adjusting for time-varying treatment and confounder history
Outcomes	The primary outcomes are cardiovascular disease and all-cause mortality. The secondary outcome is atherosclerotic cardiovascular disease. Competing events are defined as non-cardiovascular deaths before the occurrence of the outcome of interest (except for all-cause mortality).	Same as for the target trial.
Follow-up	The follow-up period starts at baseline and ends at the year of recording cardiovascular deaths, non-cardiovascular deaths, loss to follow-up, 29 years after baseline, or administrative end of follow-up on 31 December 2020, whichever occurs first.	Same as for the target trial.
Causal contrasts	Intention-to-treat effect Per-protocol effect	Observational analog of per-protocol effect
Statistical analysis	Intention-to-treat analysis Per-protocol analysis: use of parametric g-formula, an extension of standardization to adjust for time-varying interventions and confounders affected by past treatment and confounders history, with assumptions of no unmeasured confounding and measured confounder history throughout follow-up and no model misspecification. Competing risk analyses: during the follow-up period, participants can die before developing the events of interest, and the dynamic interventions may affect the risk of death.	Same per-protocol analysis with sequential emulation and adjustment for baseline covariates Same competing risk analyses

## Supplementary information

STROBE_checklist_v4_combined.

## Data Availability

Summary-level genetic data used in the applied example are publicly available from the Global Lipids Genetics Consortium (https://csg.sph.umich.edu/willer/public/glgc-lipids2021/) for LDL-C, the CARDIoGRAMplusC4D Consortium for CAD (http://www.cardiogramplusc4d.org/data-downloads/), the Neale lab for 78 clinical endpoints with ICD-10 diagnoses and LDL-C in UK Biobank (https://www.nealelab.is/uk-biobank), and the eQTLGen Consortium for expression quantitative trait loci (https://www.eqtlgen.org/phase1.html). Notably, the data from the Chinese Multi-provincial Cohort Study are not publicly available currently, so any collaboration is warmly welcomed to contact the corresponding author.

## Code availability

Codes used to implement the translational framework and reproduce the applied example results are available at https://yangzhao98.github.io/drugTargetScreen/articles/Codes_for_Paper_1.html.

## References

[1] Mensah, G. A. et al. Global burden of cardiovascular diseases and risks, 1990–2022. *J. Am. Coll. Cardiol.* **82**, 2350–2473 (2023). 38092509 10.1016/j.jacc.2023.11.007 PMC7615984

[2] Cholesterol Treatment Trialists, C. et al. The effects of lowering LDL cholesterol with statin therapy in people at low risk of vascular disease: meta-analysis of individual data from 27 randomised trials. *Lancet* **380**, 581–590 (2012). 22607822 10.1016/S0140-6736(12)60367-5 PMC3437972

[3] Ference, B. A. et al. Variation in PCSK9 and HMGCR and risk of cardiovascular disease and diabetes. *N. Engl. J. Med.* **375**, 2144–2153 (2016). 27959767 10.1056/NEJMoa1604304

[4] Di Cesare, M. et al. World Heart Report 2023: Confronting the World’s Number One Killer. *World Heart Federation: Geneva, Switzerland* (2023).

[5] Fordyce, C. B. et al. Cardiovascular drug development: is it dead or just hibernating? *J. Am. Coll. Cardiol.* **65**, 1567–1582 (2015). 25881939 10.1016/j.jacc.2015.03.016

[6] Harrison, R. K. Phase II and phase III failures: 2013-2015. *Nat. Rev. Drug Discov.* **15**, 817–818 (2016). 27811931 10.1038/nrd.2016.184

[7] Dowden, H. & Munro, J. Trends in clinical success rates and therapeutic focus. *Nat. Rev. Drug Discov.* **18**, 495–496 (2019). 31267067 10.1038/d41573-019-00074-z

[8] Sun, D., Gao, W., Hu, H. & Zhou, S. Why 90% of clinical drug development fails and how to improve it? *Acta Pharm. Sin. B* **12**, 3049–3062 (2022). 35865092 10.1016/j.apsb.2022.02.002 PMC9293739

[9] Figtree, G. A. et al. A call to action for new global approaches to cardiovascular disease drug solutions. *Circulation* **144**, 159–169 (2021). 33876947 10.1161/CIR.0000000000000981

[10] Bycroft, C. et al. The UK Biobank resource with deep phenotyping and genomic data. *Nature* **562**, 203–209 (2018). 30305743 10.1038/s41586-018-0579-z PMC6786975

[11] Jensson, B. O. et al. Actionable genotypes and their association with life span in Iceland. *N. Engl. J. Med.* **389**, 1741–1752 (2023). 37937776 10.1056/NEJMoa2300792

[12] Nagai, A. et al. Overview of the BioBank Japan Project: Study design and profile. *J. Epidemiol.* **27**, S2–S8 (2017). 28189464 10.1016/j.je.2016.12.005 PMC5350590

[13] Walters, R. G. et al. Genotyping and population characteristics of the China Kadoorie Biobank. *Cell Genom.* **3**, 100361 (2023). 37601966 10.1016/j.xgen.2023.100361 PMC10435379

[14] Mitja, I. K., et al. FinnGen: Unique genetic insights from combining isolated population and national health register data. *medRxiv*, 2022.2003.2003.22271360 (2022).

[15] Sijtsma, A. et al. Cohort Profile Update: Lifelines, a three-generation cohort study and biobank. *Int J. Epidemiol.* **51**, e295–e302 (2022). 34897450 10.1093/ije/dyab257 PMC9558073

[16] Gaziano, J. M. et al. Million Veteran Program: A mega-biobank to study genetic influences on health and disease. *J. Clin. Epidemiol.* **70**, 214–223 (2016). 26441289 10.1016/j.jclinepi.2015.09.016

[17] All of Us Research Program, I. et al. The “All of Us” Research Program. *N. Engl. J. Med.* **381**, 668–676 (2019). 31412182 10.1056/NEJMsr1809937 PMC8291101

[18] Sanderson, E. et al. Mendelian randomization. *Nat. Rev. Methods Primers* **2**, 6 (2022). 10.1038/s43586-021-00092-5 PMC7614635 37325194

[19] Zuber, V. et al. Combining evidence from Mendelian randomization and colocalization: Review and comparison of approaches. *Am. J. Hum. Genet.* **109**, 767–782 (2022). 35452592 10.1016/j.ajhg.2022.04.001 PMC7612737

[20] Gill, D. et al. Mendelian randomization for studying the effects of perturbing drug targets. *Wellcome Open Res* **6**, 16 (2021). 33644404 10.12688/wellcomeopenres.16544.1 PMC7903200

[21] Schmidt, A. F. et al. Genetic drug target validation using Mendelian randomisation. *Nat. Commun.* **11**, 3255 (2020). 32591531 10.1038/s41467-020-16969-0 PMC7320010

[22] Holmes, M. V., Richardson, T. G., Ference, B. A., Davies, N. M. & Davey Smith, G. Integrating genomics with biomarkers and therapeutic targets to invigorate cardiovascular drug development. *Nat. Rev. Cardiol.* **18**, 435–453 (2021). 33707768 10.1038/s41569-020-00493-1

[23] Burgess, S. et al. Using genetic association data to guide drug discovery and development: Review of methods and applications. *Am. J. Hum. Genet.* **110**, 195–214 (2023). 36736292 10.1016/j.ajhg.2022.12.017 PMC9943784

[24] Sun, B. B. et al. Plasma proteomic associations with genetics and health in the UK Biobank. *Nature* **622**, 329–338 (2023). 37794186 10.1038/s41586-023-06592-6 PMC10567551

[25] Nelson, M. R. et al. The support of human genetic evidence for approved drug indications. *Nat. Genet.* **47**, 856–860 (2015). 26121088 10.1038/ng.3314

[26] King, E. A., Davis, J. W. & Degner, J. F. Are drug targets with genetic support twice as likely to be approved? Revised estimates of the impact of genetic support for drug mechanisms on the probability of drug approval. *PLoS Genet.* **15**, e1008489 (2019). 31830040 10.1371/journal.pgen.1008489 PMC6907751

[27] Roberts, R. Mendelian Randomization Studies Promise to Shorten the Journey to FDA Approval. *JACC Basic Transl. Sci.* **3**, 690–703 (2018). 30456340 10.1016/j.jacbts.2018.08.001 PMC6234613

[28] Ochoa, D. et al. Human genetics evidence supports two-thirds of the 2021 FDA-approved drugs. *Nat. Rev. Drug Discov.* **21**, 551 (2022). 35804044 10.1038/d41573-022-00120-3

[29] Loos, R. J. F. 15 years of genome-wide association studies and no signs of slowing down. *Nat. Commun.* **11**, 5900 (2020). 33214558 10.1038/s41467-020-19653-5 PMC7677394

[30] Finan, C. et al. The druggable genome and support for target identification and validation in drug development. *Sci. Transl. Med*. **9**, eaag1166 (2017). 28356508 10.1126/scitranslmed.aag1166 PMC6321762

[31] Fauman, E. B. & Hyde, C. An optimal variant to gene distance window derived from an empirical definition of cis and trans protein QTLs. *BMC Bioinforma.* **23**, 169 (2022). 10.1186/s12859-022-04706-x PMC9082853 35527238

[32] Pietzner, M. et al. Mapping the proteo-genomic convergence of human diseases. *Science* **374**, eabj1541 (2021). 34648354 10.1126/science.abj1541 PMC9904207

[33] Suhre, K., McCarthy, M. I. & Schwenk, J. M. Genetics meets proteomics: perspectives for large population-based studies. *Nat. Rev. Genet.* **22**, 19–37 (2021). 32860016 10.1038/s41576-020-0268-2

[34] Dhindsa, R. S. et al. Rare variant associations with plasma protein levels in the UK Biobank. *Nature* **622**, 339–347 (2023). 37794183 10.1038/s41586-023-06547-x PMC10567546

[35] Ference, B. A. et al. Mendelian randomization study of ACLY and cardiovascular disease. *N. Engl. J. Med.* **380**, 1033–1042 (2019). 30865797 10.1056/NEJMoa1806747 PMC7612927

[36] Ference, B. A., Majeed, F., Penumetcha, R., Flack, J. M. & Brook, R. D. Effect of naturally random allocation to lower low-density lipoprotein cholesterol on the risk of coronary heart disease mediated by polymorphisms in NPC1L1, HMGCR, or both: a 2 x 2 factorial Mendelian randomization study. *J. Am. Coll. Cardiol.* **65**, 1552–1561 (2015). 25770315 10.1016/j.jacc.2015.02.020 PMC6101243

[37] Ference, B. A. et al. Association of Triglyceride-lowering LPL variants and LDL-C-lowering LDLR variants with risk of coronary heart disease. *JAMA* **321**, 364–373 (2019). 30694319 10.1001/jama.2018.20045 PMC6439767

[38] Trinder, M., Uddin, M. M., Finneran, P., Aragam, K. G. & Natarajan, P. Clinical Utility of Lipoprotein(a) and LPA genetic risk score in risk prediction of incident atherosclerotic cardiovascular disease. *JAMA Cardiol.* **6**, 1–9 (2020). 10.1001/jamacardio.2020.5398 PMC7539232 33021622

[39] Davey Smith, G. & Ebrahim, S. What can mendelian randomisation tell us about modifiable behavioural and environmental exposures? *BMJ* **330**, 1076–1079 (2005). 15879400 10.1136/bmj.330.7499.1076 PMC557238

[40] Burgess, S., Butterworth, A., Malarstig, A. & Thompson, S. G. Use of Mendelian randomisation to assess potential benefit of clinical intervention. *BMJ* **345**, e7325 (2012). 23131671 10.1136/bmj.e7325

[41] Uffelmann, E. et al. Genome-wide association studies. *Nat. Rev. Methods Prim.* **1**, 59 (2021).

[42] Panagiotou, O. A. & Ioannidis, J. P. What should the genome-wide significance threshold be? Empirical replication of borderline genetic associations. *Int J. Epidemiol.* **41**, 273–286 (2012). 22253303 10.1093/ije/dyr178

[43] Storey, J. D. & Tibshirani, R. Statistical significance for genomewide studies. *Proc. Natl Acad. Sci.* **100**, 9440–9445 (2003). 12883005 10.1073/pnas.1530509100 PMC170937

[44] Menyhart, O., Weltz, B. & Győrffy, B. MultipleTesting.com: A tool for life science researchers for multiple hypothesis testing correction. *PLoS One* **16**, e0245824 (2021). 34106935 10.1371/journal.pone.0245824 PMC8189492

[45] John, D. S. The positive false discovery rate: a Bayesian interpretation and the q-value. *Ann. Stat.* **31**, 2013–2035 (2003).

[46] Wang, J. et al. Causal inference for heritable phenotypic risk factors using heterogeneous genetic instruments. *PLoS Genet* **17**, e1009575 (2021). 34157017 10.1371/journal.pgen.1009575 PMC8301661

[47] Iong, D., Zhao, Q. & Chen, Y. A Latent mixture model for heterogeneous causal mechanisms in Mendelian Randomization. *Ann Appl Stat* **1**, 966–990 (2024).

[48] Gao, Z., Hastie, T. & Zhao, Q. PathGPS: Discover shared genetic architecture using biobank data. *Biometrics* **80**, ujae060 (2024). 39005072 10.1093/biomtc/ujae060 PMC11247175

[49] Dale, C. E. et al. Causal associations of adiposity and body fat distribution with coronary heart disease, stroke subtypes, and Type 2 Diabetes Mellitus: A Mendelian randomization analysis. *Circulation* **135**, 2373–2388 (2017). 28500271 10.1161/CIRCULATIONAHA.116.026560 PMC5515354

[50] Ross, S. et al. Mendelian randomization analysis supports the causal role of dysglycaemia and diabetes in the risk of coronary artery disease. *Eur. Heart J.* **36**, 1454–1462 (2015). 25825043 10.1093/eurheartj/ehv083

[51] Gregg, E. W. et al. Association of the magnitude of weight loss and changes in physical fitness with long-term cardiovascular disease outcomes in overweight or obese people with type 2 diabetes: a post-hoc analysis of the Look AHEAD randomised clinical trial. *Lancet Diab. Endocrinol.* **4**, 913–921 (2016). 10.1016/S2213-8587(16)30162-0 PMC5094846 27595918

[52] Seidelmann, S. B. et al. Genetic variants in SGLT1, glucose tolerance, and cardiometabolic risk. *J. Am. Coll. Cardiol.* **72**, 1763–1773 (2018). 30286918 10.1016/j.jacc.2018.07.061 PMC6403489

[53] Zinman, B. et al. Empagliflozin, cardiovascular outcomes, and mortality in Type 2 diabetes. *N. Engl. J. Med.* **373**, 2117–2128 (2015). 26378978 10.1056/NEJMoa1504720

[54] Wanner, C. et al. Empagliflozin and progression of kidney disease in Type 2 diabetes. *N. Engl. J. Med.* **375**, 323–334 (2016). 27299675 10.1056/NEJMoa1515920

[55] Blauw, L. L. et al. CETP (Cholesteryl Ester Transfer Protein) Concentration: A genome-wide association study followed by mendelian randomization on coronary artery disease. *Circ. Genom. Precis Med* **11**, e002034 (2018). 29728394 10.1161/CIRCGEN.117.002034

[56] Thompson, A. et al. Association of cholesteryl ester transfer protein genotypes with CETP mass and activity, lipid levels, and coronary risk. *JAMA* **299**, 2777–2788 (2008). 18560005 10.1001/jama.299.23.2777

[57] Ference, B. A. et al. Association of genetic variants related to CETP inhibitors and statins with Lipoprotein levels and cardiovascular risk. *JAMA* **318**, 947–956 (2017). 28846118 10.1001/jama.2017.11467 PMC5710502

[58] Wang, G., Sarkar, A., Carbonetto, P. & Stephens, M. A simple new approach to variable selection in regression, with application to genetic fine mapping. *J. R. Stat. Soc. Ser. B: Stat. Methodol.* **82**, 1273–1300 (2020). 10.1111/rssb.12388 PMC10201948 37220626

[59] Wallace, C. A more accurate method for colocalisation analysis allowing for multiple causal variants. *PLoS Genet* **17**, e1009440 (2021). 34587156 10.1371/journal.pgen.1009440 PMC8504726

[60] GTEx Consortium. The GTEx Consortium atlas of genetic regulatory effects across human tissues. *Science* **369**, 1318–1330 (2020). 10.1126/science.aaz1776 PMC7737656 32913098

[61] Vosa, U. et al. Large-scale cis- and trans-eQTL analyses identify thousands of genetic loci and polygenic scores that regulate blood gene expression. *Nat. Genet.* **53**, 1300–1310 (2021). 34475573 10.1038/s41588-021-00913-z PMC8432599

[62] Freedman, M. L. et al. Principles for the post-GWAS functional characterization of cancer risk loci. *Nat. Genet.* **43**, 513–518 (2011). 21614091 10.1038/ng.840 PMC3325768

[63] Yao, C. et al. Genome-wide mapping of plasma protein QTLs identifies putatively causal genes and pathways for cardiovascular disease. *Nat. Commun.* **9**, 3268 (2018). 30111768 10.1038/s41467-018-05512-x PMC6093935

[64] Eldjarn, G. H. et al. Large-scale plasma proteomics comparisons through genetics and disease associations. *Nature* **622**, 348–358 (2023). 37794188 10.1038/s41586-023-06563-x PMC10567571

[65] Koprulu, M. et al. Proteogenomic links to human metabolic diseases. *Nat. Metab.* **5**, 516–528 (2023). 36823471 10.1038/s42255-023-00753-7 PMC7614946

[66] Yang, G. & Schooling, C. M. Genetically mimicked effects of ASGR1 inhibitors on all-cause mortality and health outcomes: a drug-target Mendelian randomization study and a phenome-wide association study. *BMC Med.* **21**, 235 (2023). 37400795 10.1186/s12916-023-02903-w PMC10318778

[67] Luo, S. et al. Assessing the safety of lipid-modifying medications among Chinese adolescents: a drug-target Mendelian randomization study. *BMC Med.* **21**, 410 (2023). 37904165 10.1186/s12916-023-03115-y PMC10617134

[68] Hernan, M. A. & Robins, J. M. Using big data to emulate a target trial when a randomized trial Is not available. *Am. J. Epidemiol.* **183**, 758–764 (2016). 26994063 10.1093/aje/kwv254 PMC4832051

[69] Hernan, M. A., Wang, W. & Leaf, D. E. Target trial emulation: A framework for causal inference from observational data. *JAMA* **328**, 2446–2447 (2022). 36508210 10.1001/jama.2022.21383

[70] Hernan, M. A. Methods of public health research - Strengthening causal inference from observational data. *N. Engl. J. Med.* **385**, 1345–1348 (2021). 34596980 10.1056/NEJMp2113319

[71] Hernán, M. A. & Robins, J. M. Per-Protocol Analyses of Pragmatic Trials. *N. Engl. J. Med.* **377**, 1391–1398 (2017). 28976864 10.1056/NEJMsm1605385

[72] Hernan, M. A., Sauer, B. C., Hernandez-Diaz, S., Platt, R. & Shrier, I. Specifying a target trial prevents immortal time bias and other self-inflicted injuries in observational analyses. *J. Clin. Epidemiol.* **79**, 70–75 (2016). 27237061 10.1016/j.jclinepi.2016.04.014 PMC5124536

[73] Matthews, A. A., Danaei, G., Islam, N. & Kurth, T. Target trial emulation: applying principles of randomised trials to observational studies. *BMJ* **378**, e071108 (2022). 36041749 10.1136/bmj-2022-071108

[74] Dickerman, B. A. et al. Comparative Effectiveness of BNT162b2 and mRNA-1273 Vaccines in U.S. Veterans. *N. Engl. J. Med.* **386**, 105–115 (2021). 34942066 10.1056/NEJMoa2115463 PMC8693691

[75] Barda, N. et al. Effectiveness of a third dose of the BNT162b2 mRNA COVID-19 vaccine for preventing severe outcomes in Israel: an observational study. *Lancet* **398**, 2093–2100 (2021). 34756184 10.1016/S0140-6736(21)02249-2 PMC8555967

[76] Magen, O. et al. Fourth dose of BNT162b2 mRNA Covid-19 vaccine in a nationwide setting. *N. Engl. J. Med.* **386**, 1603–1614 (2022). 35417631 10.1056/NEJMoa2201688 PMC9020581

[77] Moreira, E. D. et al. Safety and efficacy of a third dose of BNT162b2 Covid-19 vaccine. *N. Engl. J. Med.* **386**, 1910–1921 (2022). 35320659 10.1056/NEJMoa2200674 PMC9006787

[78] Lawlor, D. A., Tilling, K. & Davey Smith, G. Triangulation in aetiological epidemiology. *Int J. Epidemiol.* **45**, 1866–1886 (2016). 28108528 10.1093/ije/dyw314 PMC5841843

[79] Munafo, M. R. & Davey Smith, G. Robust research needs many lines of evidence. *Nature* **553**, 399–401 (2018). 29368721 10.1038/d41586-018-01023-3

[80] O’Donoghue, M. L. et al. Small interfering RNA to reduce Lipoprotein(a) in cardiovascular disease. *N. Engl. J. Med.* **387**, 1855–1864 (2022). 36342163 10.1056/NEJMoa2211023

[81] Malick, W. A., Goonewardena, S. N., Koenig, W. & Rosenson, R. S. Clinical trial design for Lipoprotein(a)-lowering therapies: JACC Focus Seminar 2/3. *J. Am. Coll. Cardiol.* **81**, 1633–1645 (2023). 37076218 10.1016/j.jacc.2023.02.033

[82] Nurmohamed, N. S., Navar, A. M. & Kastelein, J. J. P. New and emerging therapies for reduction of LDL-Cholesterol and Apolipoprotein B: JACC Focus Seminar 1/4. *J. Am. Coll. Cardiol.* **77**, 1564–1575 (2021). 33766264 10.1016/j.jacc.2020.11.079

[83] Graham, S. E. et al. The power of genetic diversity in genome-wide association studies of lipids. *Nature* **600**, 675–679 (2021). 34887591 10.1038/s41586-021-04064-3 PMC8730582

[84] Nikpay, M. et al. A comprehensive 1000 Genomes–based genome-wide association meta-analysis of coronary artery disease. *Nat. Genet.* **47**, 1121–1130 (2015). 26343387 10.1038/ng.3396 PMC4589895

[85] Liu, J. et al. Predictive value for the Chinese population of the Framingham CHD risk assessment tool compared with the Chinese Multi-Provincial Cohort Study. *JAMA* **291**, 2591–2599 (2004). 15173150 10.1001/jama.291.21.2591

[86] Qi, Y. et al. Long-term cardiovascular risk associated with Stage 1 hypertension defined by the 2017 ACC/AHA Hypertension Guideline. *J. Am. Coll. Cardiol.* **72**, 1201–1210 (2018). 30189996 10.1016/j.jacc.2018.06.056

[87] Hemani, G. et al. The MR-Base platform supports systematic causal inference across the human phenome. *Elife* **7**, e34408 (2018). 29846171 10.7554/eLife.34408 PMC5976434

[88] Staley, J. R. et al. PhenoScanner: a database of human genotype-phenotype associations. *Bioinformatics* **32**, 3207–3209 (2016). 27318201 10.1093/bioinformatics/btw373 PMC5048068

[89] Kamat, M. A. et al. PhenoScanner V2: an expanded tool for searching human genotype-phenotype associations. *Bioinformatics* **35**, 4851–4853 (2019). 31233103 10.1093/bioinformatics/btz469 PMC6853652

[90] McLaren, W. et al. The Ensembl Variant Effect Predictor. *Genome Biol.* **17**, 122 (2016). 27268795 10.1186/s13059-016-0974-4 PMC4893825

[91] Chen, S. et al. A genome-wide mutational constraint map quantified from variation in 76,156 human genomes. *bioRxiv*, 2022.2003.2020.485034 (2022).

[92] Zhou, H. et al. FAVOR: functional annotation of variants online resource and annotator for variation across the human genome. *Nucleic Acids Res* **51**, D1300–d1311 (2023). 36350676 10.1093/nar/gkac966 PMC9825437

[93] Zou, Y., Carbonetto, P., Wang, G. & Stephens, M. Fine-mapping from summary data with the “Sum of Single Effects” model. *PLoS Genet* **18**, e1010299 (2022). 35853082 10.1371/journal.pgen.1010299 PMC9337707

[94] Foley, C. N. et al. A fast and efficient colocalization algorithm for identifying shared genetic risk factors across multiple traits. *Nat. Commun.* **12**, 764 (2021). 33536417 10.1038/s41467-020-20885-8 PMC7858636

[95] Croft, D. et al. Reactome: a database of reactions, pathways and biological processes. *Nucleic Acids Res.* **39**, D691–D697 (2011). 21067998 10.1093/nar/gkq1018 PMC3013646

[96] Smith, G. D. & Ebrahim, S. Mendelian randomization’: Can genetic epidemiology contribute to understanding environmental determinants of disease? *Int J. Epidemiol.* **32**, 1–22 (2003). 12689998 10.1093/ije/dyg070

[97] VanderWeele, T. J., Tchetgen Tchetgen, E. J., Cornelis, M. & Kraft, P. Methodological challenges in mendelian randomization. *Epidemiology* **25**, 427–435 (2014). 24681576 10.1097/EDE.0000000000000081 PMC3981897

[98] Schooling, C. M. et al. Use of multivariable Mendelian randomization to address biases due to competing risk before recruitment. *Front. Genet.* **11**, 610852 (2020). 33519914 10.3389/fgene.2020.610852 PMC7845663

[99] Yang, Z., Schooling, C. M. & Kwok, M. K. Credible Mendelian randomization studies in the presence of selection bias using control exposures. *Front. Genet.* **12**, 729326 (2021). 34899831 10.3389/fgene.2021.729326 PMC8652250

[100] Burgess, S. & Thompson, S. G. Multivariable Mendelian randomization: the use of pleiotropic genetic variants to estimate causal effects. *Am. J. Epidemiol.* **181**, 251–260 (2015). 25632051 10.1093/aje/kwu283 PMC4325677

[101] Uhlén, M. et al. Tissue-based map of the human proteome. *Science* **347**, 1260419 (2015). 25613900 10.1126/science.1260419

[102] Buniello, A. et al. The NHGRI-EBI GWAS Catalog of published genome-wide association studies, targeted arrays and summary statistics 2019. *Nucleic Acids Res.* **47**, D1005–d1012 (2019). 30445434 10.1093/nar/gky1120 PMC6323933

[103] Uffelmann, E. & Posthuma, D. Emerging methods and resources for biological interrogation of neuropsychiatric polygenic signal. *Biol. Psychiatry* **89**, 41–53 (2021). 32736792 10.1016/j.biopsych.2020.05.022

[104] Abdellaoui, A., Yengo, L., Verweij, K. J. H. & Visscher, P. M. 15 years of GWAS discovery: Realizing the promise. *Am. J. Hum. Genet.* **110**, 179–194 (2023). 36634672 10.1016/j.ajhg.2022.12.011 PMC9943775

[105] Benner, C. et al. FINEMAP: efficient variable selection using summary data from genome-wide association studies. *Bioinformatics* **32**, 1493–1501 (2016). 26773131 10.1093/bioinformatics/btw018 PMC4866522

[106] Hormozdiari, F. et al. Colocalization of GWAS and eQTL signals detects target genes. *Am. J. Hum. Genet.* **99**, 1245–1260 (2016). 27866706 10.1016/j.ajhg.2016.10.003 PMC5142122

[107] Weeks, E. M. et al. Leveraging polygenic enrichments of gene features to predict genes underlying complex traits and diseases. *Nat. Genet.* **55**, 1267–1276 (2023). 37443254 10.1038/s41588-023-01443-6 PMC10836580

[108] Bock, C. et al. High-content CRISPR screening. *Nat. Rev. Methods Prim.* **2**, 8 (2022). 10.1038/s43586-022-00098-7 PMC10200264 37214176

[109] Burgess, S., Butterworth, A. & Thompson, S. G. Mendelian randomization analysis with multiple genetic variants using summarized data. *Genet. Epidemiol.* **37**, 658–665 (2013). 24114802 10.1002/gepi.21758 PMC4377079

[110] Bowden, J., Davey Smith, G., Haycock, P. C. & Burgess, S. Consistent estimation in Mendelian randomization with some invalid instruments using a weighted median estimator. *Genet. Epidemiol.* **40**, 304–314 (2016). 27061298 10.1002/gepi.21965 PMC4849733

[111] Hartwig, F. P., Davey Smith, G. & Bowden, J. Robust inference in summary data Mendelian randomization via the zero modal pleiotropy assumption. *Int J. Epidemiol.* **46**, 1985–1998 (2017). 29040600 10.1093/ije/dyx102 PMC5837715

[112] Bowden, J., Davey Smith, G. & Burgess, S. Mendelian randomization with invalid instruments: Effect estimation and bias detection through Egger regression. *Int J. Epidemiol.* **44**, 512–525 (2015). 26050253 10.1093/ije/dyv080 PMC4469799

[113] Bowden, J. et al. Improving the accuracy of two-sample summary-data Mendelian randomization: moving beyond the NOME assumption. *Int J. Epidemiol.* **48**, 728–742 (2019). 30561657 10.1093/ije/dyy258 PMC6659376

