# Author Correction: A translational framework of genoproteomic studies for cardiovascular drug discovery

**DOI:** 10.1038/s44325-025-00067-5

**Published:** 2025-06-19

**Authors:** Zhao Yang, Jie V. Zhao, Yue Qi, Xuan Deng, Zhili Ji, Jing Liu

**Affiliations:** 1 Center for Clinical and Epidemiologic Research, Beijing Anzhen Hospital, Capital Medical University, Beijing Institute of Heart, Lung and Blood Vessel Diseases, Beijing, People’s Republic of China; 2 School of Public Health, Li Ka Shing Faculty of Medicine, The University of Hong Kong, 7 Sassoon Road, Pokfulam, Hong Kong SAR, China; 3 Beijing Anzhen Hospital, Capital Medical University, Beijing, China

**Keywords:** Cardiovascular genetics, Interventional cardiology

Correction to: *npj Cardiovascular Health* 10.1038/s44325-024-00015-9, published online 6 August 2024

In this article the Competing Interests statement was incorrect and should have been ‘Author Jing Liu is Editor-in-Chief of *npj Cardiovascular Health*. Jing Liu was not involved in the journal’s review of, or decisions related to, this manuscript.’ The original article has been corrected.

